# The oncological safety in minimally invasive *versus* open distal pancreatectomy for pancreatic ductal adenocarcinoma: a systematic review and meta-analysis

**DOI:** 10.1038/s41598-018-37617-0

**Published:** 2019-02-04

**Authors:** Du-Jiang Yang, Jun-Jie Xiong, Hui-Min Lu, Yi Wei, Ling Zhang, Shan Lu, Wei-Ming Hu

**Affiliations:** 1 Department of Pancreatic Surgery, West China Hospital, Sichuan University, No. 37, Guoxue Alley, Chengdu, 610041 Sichuan Province China; 2 Department of Transportation Center, West China Hospital, Sichuan University, No. 37, Guoxue Alley, Chengdu, 610041 Sichuan Province China

## Abstract

The safety of minimally invasive distal pancreatectomy (MIDP) and open distal pancreatectomy (ODP) regarding oncological outcomes of pancreatic ductal adenocarcinoma (PDAC) remains inconclusive. Therefore, the aim of this study was to examine the oncological safety of MIDP and ODP for PDAC. Major databases including PubMed, Embase, Science Citation Index Expanded, and the Cochrane Library were searched for studies comparing outcomes in patients undergoing MIDP and ODP for PDAC from January 1994 to August 2018. In total, 11 retrospective comparative studies with 4829 patients (MIDP: 1076, ODP: 3753) were included. The primary outcome was long-term survival, including 3-year overall survival (OS) and 5-year OS. The 3-year OS (hazard ratio (HR): 1.03, 95% confidence interval (CI): 0.89, 1.21; *P* = 0.66) and 5-year OS (HR: 0.91, 95% CI: 0.65, 1.28; *P* = 0.59) showed no significant differences between the two groups. Furthermore, the positive surgical margin rate (weighted mean difference (WMD): 0.71, 95% CI: 0.56, 0.89, *P* = 0.003) was lower in the MIDP group. However, patients in the MIDP group had less intraoperative blood loss (WMD: −250.03, 95% CI: −359.68, −140.39; *P* < 0.00001), a shorter hospital stay (WMD: −2.76, 95% CI: −3.73, −1.78; *P* < 0.00001) and lower morbidity (OR: 0.57, 95% CI: 0.46, 0.71; *P* < 0.00001) and mortality (OR: 0.50, 95% CI: 0.31, 0.81, *P* = 0.005) than patients in the OD*P* group. The limited evidence suggested that MIDP might be safer with regard to oncological outcomes in PDAC patients. Therefore, future high-quality studies are needed to examine the oncological safety of MIDP.

## Introduction

Pancreatic cancer is the fourth leading cause of cancer-related death among men and women in the USA and will become the second most deadly cancer in the near future^1,2^. Surgical resection remains the main treatment for pancreatic cancer^3^. The first minimally invasive distal pancreatectomy (MIDP) was reported in 1994. With the development of advanced technology, minimally invasive techniques have increasingly been used in pancreatic surgery^4^. MIDP is regarded as a safe and feasible procedure for pancreatic surgery^5–7^. However, one study showed that almost one-third of European pancreatic surgeons considered MIDP inferior to open distal pancreatectomy (ODP) in terms of oncological outcomes^8^. Furthermore, almost 21% of pancreatic surgeons considered the minimally invasive approach contraindicated for pancreatic ductal adenocarcinoma (PDAC)^9^. Moreover, some studies have shown that MIDP was not usually performed in clinical practice^10^.

Previously, a Cochrane review was published that describes all types of pancreatic cancer^11^. However, the oncological safety of MIDP for PDAC is still not clear. At present, some high-quality studies focusing on PDAC have been published, including one propensity score-matched study performed in Europe^12^. Thus, we conducted a systematic review and meta-analysis to evaluate the oncological safety of MIDP for PDAC.

## Materials and Methods

### Literature search

A systematic literature search was performed in PubMed, Embase, Science Citation Index Expanded, and the Cochrane Library to identify and retrieve studies published from January 1994 to August 2018 that examined distal pancreatectomy for PDAC (last search on August 8, 2018). The following medical search headings were used: (“Pancreatic Cancer” or “Pancreatic ductal adenocarcinoma” or “Pancreatic adenocarcinoma”) and (“Left pancreatectomy” or “Distal Pancreatectomy” or “Pancreatectomy”) and (“Laparoscopy” or “Laparoscopic-assisted” or “Laparoscopic” or “Robotic” or “Robot-assisted” or “Mini-invasive” or “Minimally invasive”). The language of full text articles was limited to English. In addition, the references of all selected articles were screened for any potential eligible studies.

### Study selection

Studies were included in the meta-analysis if they met the following criteria: (1) human study; (2) published in English; (3) if studies were reported by the same institution (and/or authors), the study with either the larger sample size or higher quality was included. Studies meeting the following criteria were excluded: (1) case reports, letters, editorials, expert opinions and abstracts; (2) benign or other malignant tumors were included without reporting PDAC separately.

### Qualitative assessment of the studies selected

The risk of bias of included nonrandomized studies was evaluated according to the risk of bias in nonrandomized studies of interventions (ROBINS-I) tool^13^.

### Data extraction and synthesis

Each study was evaluated by two independent reviewers (Du-Jiang Yang and Jun-Jie Xiong) for inclusion or exclusion from the review. Disagreements between the reviewers were resolved by consensus and by consultation with a third reviewer (Hui-Min Lu) when necessary. Data were collected by two independent researchers using standardized forms. The study characteristics, quality assessment, and intraoperative and postoperative outcomes were included. The means of outcomes were used for the meta-analysis unless otherwise mentioned. Furthermore, the means and standard deviations or medians and ranges were reported^14^.

The following data were extracted from each study: author, year, country, study design, study duration, number of patients, age, sex, body mass index, tumor size, operation time, intraoperative blood loss, hospital stay, morbidity, postoperative pancreatic fistula (POPF) occurrence, mortality, positive surgical margin rate, lymph nodes harvested, perineural and lymphovascular invasion, multivisceral resection, positive lymph nodes, recurrence, adjuvant therapy and follow-up time.

### Outcomes of interest and definitions

Minimally invasive was defined as a laparoscopic, robotic, laparoscopic-assisted or robot-assisted procedure. The primary outcome was overall survival (OS) time, which was defined as the time from the operation until death or the final follow-up evaluation. The secondary outcomes included operative time, intraoperative blood loss, hospital stay, morbidity, POPF occurrence, mortality, positive surgical margin, lymph nodes harvested, recurrence, perineural and lymphovascular invasion, multivisceral resection, positive lymph nodes, and adjuvant therapy. The operative time was defined as the interval from incision to suturing of the skin. Intraoperative blood loss was defined as the blood loss during surgery. The hospital stay was defined as the length from patient admission to discharge from the hospital. Morbidity was defined as all complications that occurred during the hospital stay or within 90 days after surgery. POPF occurrence was defined according to the International Study Group of Pancreatic Fistula (ISGPF)^15^. Mortality was defined as the number of deaths occurring during hospitalization or within 30 days after surgery. A positive surgical margin was defined as tumor in the transection and circumferential margins with a distance from the margin to the tumor of <1 mm^12,16,17^ or <0 mm^18,19^. Lymph nodes harvested was defined as the number of lymph nodes obtained during the operation. Recurrence was defined as local recurrence or distant metastasis. Adjuvant therapy was defined as the number of patients who received adjuvant therapy including postoperative radiation or chemotherapy.

### Statistical analysis

A meta-analysis was performed using Review Manager Version 5.3 software (The Cochrane Collaboration, Oxford, UK). Variables are expressed as weighted mean differences (WMDs) or odds ratios (ORs) as appropriate, with their corresponding 95% confidence interval (CI). For continuous and categorical variables, treatment effects are expressed as WMDs and ORs with corresponding 95% CIs. For the survival analysis, we extracted data from the survival curve using a method reported in a previous study, and hazard ratios (HRs) were used for the quantitative analysis^20^. A Chi-square test was used to assess heterogeneity, with *P* < 0.1 considered significant. *I*^2^ values were used to evaluate statistical heterogeneity; an *I*^2^ value of 50% or more indicated the presence of heterogeneity^21^. The fixed-effects model was initially used for all outcomes, while the random-effects model was used if the test suggested rejection of the assumption of homogeneity^22^. Descriptive methods were used if the data were inappropriate for meta-analysis. A sensitivity analysis was performed by removing individual studies from the data set and analyzing the effect on the overall results to identify sources of heterogeneity. A funnel plot was constructed to evaluate potential publication bias based on the OS and positive surgical margin rate^23^.

## Results

### Description of the included studies

**Figure 1:**
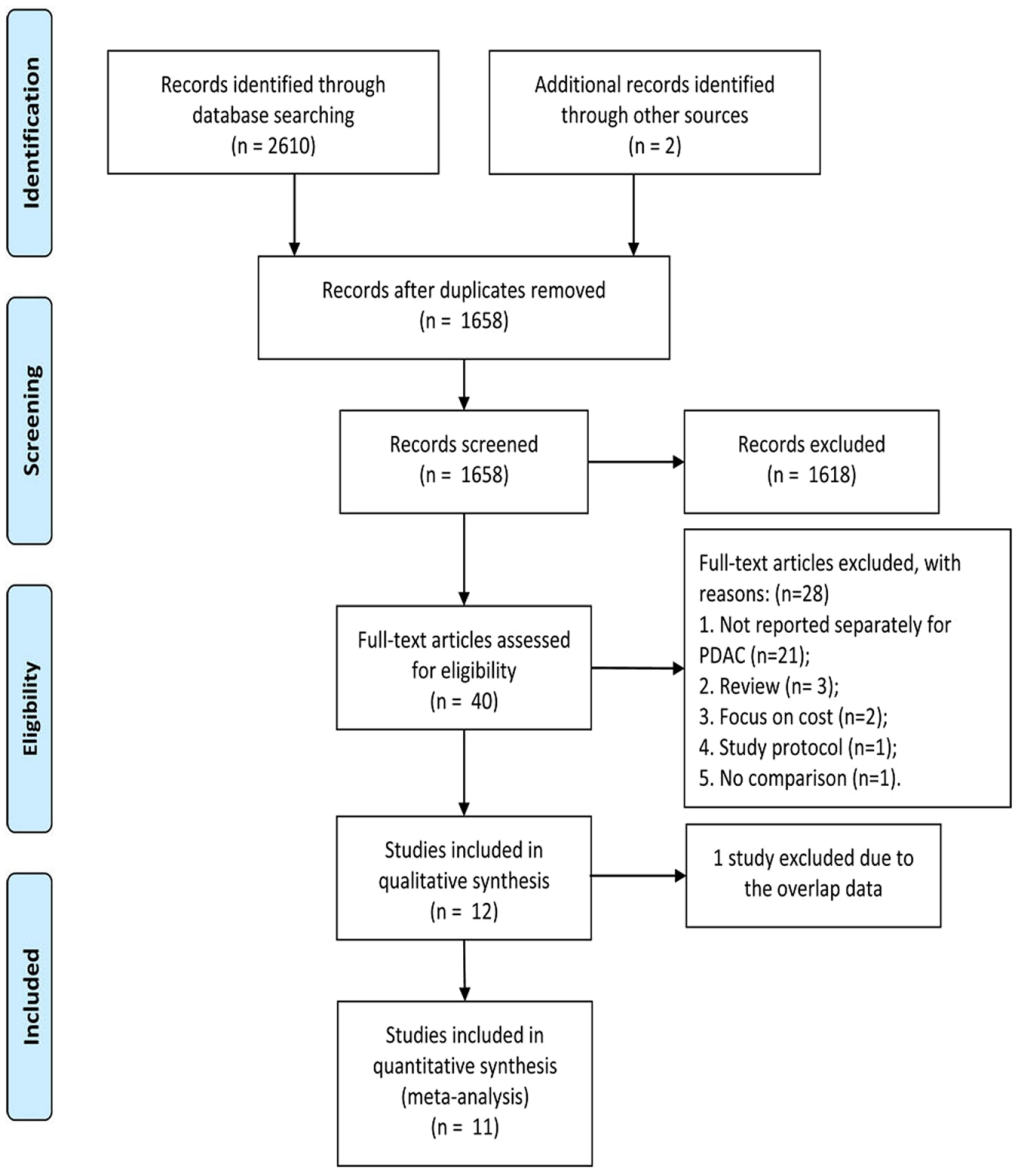
Flow chart for study search (PRISMA diagram). (PDAC = pancreatic ductal adenocarcinoma).

The reporting of this systematic review was in accordance with the PRISMA Statement^24^. A flow diagram of the study is shown in Fig. 1. In total, 2612 studies were identified from the electronic databases, and 954 studies were excluded because they were duplicate publications. Finally, the full texts of 40 studies^11,12,16–19,25–58^ were screened for eligibility; however, 28 studies were excluded for various reasons^11,25,27–35,38,39,41–43,45–48,50–55,57,58^ (Supplementary Materials). Only 12 studies^12,16–19,26,36,37,40,44,49,56^ were included for further analysis. However, two studies^36,49^ originated from the National Cancer Database (NCDB). Finally, the study by Kantor^49^ was excluded because of irrelevant data. In total, 11 studies^12,16–19,26,36,37,40,44,56^ with 4829 patients (MIDP: 1076, ODP: 3753) were included in the meta-analysis. The study characteristics are shown in Table 1. The studies originated from the United States (4 studies)^18,26,36,44^, China (2 studies)^17,56^, Korea^37^, the UK^16^, France^40^, Italy^19^, and Europe^12^. The tumor size, which was reported in 10 studies, was larger in patients who underwent ODP than in those who underwent MIDP (WMD: −0.45, 95% CI: −0.85, −0.05; *P* = 0.03). All included studies were retrospective comparative studies. The risk of bias in included studies was evaluated by the ROBINS-I tool (Table 2). Based on the ROBINS-I assessment, two studies^12,37^ were considered to have low risk, four studies^17,18,26,40^ were considered to have moderate risk, and five studies^16,19,36,44,56^ were considered to have serious risk of bias. Perioperative and tumor outcomes are shown in Tables 3 and 4. The results of the meta-analysis are shown in Table 5 and the Supplementary Materials.

**Table 1:** Characteristics of the included studies.

Author	Year	Country	Study duration	Design	No of patients		Age (year)		Sex (F/M)		BMI (kg/m^2^)		Tumor size (cm)	
					MIDP	ODP	MIDP	ODP	MIDP	ODP	MIDP	ODP	MIDP	ODP
Kooby^18^	2010	USA	2000–2008	Retro	23	70	64.6 ± 12.3	65.9 ± 11.1	12/11	43/27	28.5 ± 5.7	25.8 ± 4.6	3.6 ± 1.3	3.5 ± 1.4
Magge^26^	2013	USA	2002–2010	Retro	28	34	67 + 11.6	66 ± 11.7	19/9	21/13	26.7 ± 1.3	26.5 ± 0.7	3.7 ± 1.3	4.5 ± 1.9
Rehman^16^	2014	UK	2008–2011	Retro	8	14	64.2	64	3/8	13/1	NR	NR	2.4 ± 0.9	3.4 ± 1.4
Sharpe^36^	2015	USA	2010–2011	Retro	144	625	67.7 ± 10.1	65.6 ± 10.5	NR	NR	NR	NR	3.7 ± 1.9	4.2 ± 3.2
Shin^37^	2015	Korea	2006–2013	PSM	70	80	61 ± 7.8	65 ± 6	23/47	32/48	24.1 ± 2.1	23.1 ± 2.2	3 ± 1.35	3.5 ± 2.25
Sulpice^40^	2015	French	2007–2012	Retro	347	2406	60.6 ± 14.7	64.5 ± 12.0	196/151	1236/1170	NR	NR	NR	NR
Zhang^17^	2015	China	2003–2013	Retro	17	34	60 ± 7.75	64 ± 9	6/11	15/19	23.4 ± 4.7	23.7 ± 2.4	3.5 ± 0.8	3.9 ± 0.9
Stauffer^19^	2016	Italy	1995–2014	Retro	44	28	72 ± 5.8	67.3 ± 6.8	18/26	12/16	28.3 ± 7.7	26.1 ± 4.3	3.6 ± 1.2	4.5 ± 2.5
Bauman^44^	2017	USA	2005–2014	Retro	33	46	66 ± 2	66 ± 2	16/17	28/18	26.2 ± 0.8	27.8 ± 0.9	3.3 ± 0.3	4.0 ± 0.4
Hilst^12^	2017	European	2007–2015	PSM	340	340	68 ± 10	68 ± 10	164/176	157/183	25 ± 3.7	25 ± 4.4	3.5 ± 1.5	3 ± 1.6
Zhang^56^	2017	China	2010–2014	Retro	22	76	55.2 ± 13.1	59.8 ± 9.0	13/9	46/30	23.9 ± 2.7	23.7 ± 3.3	3.6 ± 1.3	4.4 ± 1.4

No = Number; F = Female; M = Man; BMI = Body mass index; MIDP = Minimally invasive distal pancreatectomy; ODP = Open distal pancreatectomy; Retro = Retrospective; PSM = Propensity score matching; NR = Not report; Data shown represents mean ± standard deviation.

**Table 2:** Risk of bias for included studies.

ROBINS-I	Kooby^18^	Magge^26^	Rehman^16^	Sharpe^36^	Shin^37^	Sulpice^40^	Zhang^17^	Stauffer^19^	Bauman^44^	Hilst^12^	Zhang^56^
Bias due to confounding	L	L	S	S	L	M	L	S	S	L	S
Bias in selection of participants into the study	M	L	L	L	L	L	L	L	L	L	L
Bias in measurement of interventions	L	L	L	L	L	M	L	L	L	L	L
Bias due to departures from intended interventions	M	M	M	L	L	M	M	M	M	L	M
Bias due to missing data	L	L	L	L	L	L	L	L	L	L	L
Bias in measurement of outcomes	L	L	L	L	L	L	L	L	M	L	L
Bias in selection of the reported result	L	M	M	M	L	M	M	M	M	L	M
Overall	M	M	S	S	L	M	M	S	S	L	S

ROBINS-I = Risk of bias in non-randomized studies of interventions; L = Lower; M = Moderate; S = Serious.

**Table 3:** Perioperative outcomes.

Author	Operation time(min)		Intraoperative blood loss(mL)		Hospital stay(days)		Morbidity		POPF		Mortality	
	MIDP	ODP	MIDP	ODP	MIDP	ODP	MIDP	ODP	MIDP	ODP	MIDP	ODP
Kooby^18^	238.4 ± 68	216 ± 69	422 ± 473	751 ± 853	7.4 ± 3.4	9.4 ± 4.7	NR	NR	NR	NR	0	2
Magge^26^	294 ± 126.9	317 ± 134.1	290 ± 317.5	570 ± 466.5	6 ± 2	8 ± 2.2	11	17	6	10	NR	NR
Rehman^16^	396.5 ± 58.5	287 ± 60	349.25 ± 47.5	686.3 ± 288.8	8.75 ± 2.25	12.75 ± 3.75	3	3	2	3	1	1
Sharpe^36^	NR	NR	NR	NR	6.8 ± 4.6	8.9 ± 7.5	NR	NR	NR	NR	0	10
Shin^37^	239 ± 45.3	254 ± 76.3	NR	NR	9 ± 4	12 ± 20	NR	NR	13	8	0	1
Sulpice^40^	NR	NR	NR	NR	14.9 ± 8.9	19.6 ± 14.6	117	1142	NR	NR	9	135
Zhang^17^	190 ± 72.5	245 ± 66.25	50 ± 117.5	400 ± 950	13 ± 4.5	15.5 ± 8.5	NR	NR	9	16	0	1
Stauffer^19^	254 ± 70.3	266 ± 68.7	332 ± 440	874 ± 541.7	5.1 ± 2.5	9.4 ± 5.3	6	7	6	2	1	0
Bauman^44^	234 ± 12	252 ± 12	310 ± 68	597 ± 95	7.6 ± 1.4	9 ± 0.7	17	32	8	16	1	7
Hilst^12^	240 ± 85.18	230 ± 80	200 ± 251.9	300 ± 259.3	8 ± 4.4	9 ± 5.2	NR	NR	65	67	7	8
Zhang^56^	188 ± 39	160 ± 35	210 ± 130	240 ± 120	NR	NR	NR	NR	8	19	0	0

POPF = Postoperative pancreatic fistula; MIDP = Minimally invasive distal pancreatectomy; ODP = Open distal pancreatectomy; NR = Not reported; Data shown represents mean ± standard deviation.

**Table 4:** Tumor outcomes.

Author	Positive surgical margin		Lymph nodes harvested		Recurrence		Adjuvant chemotherapy		Perineural and lymfovascular invasion		Positive lymphnodes		Vascular resection		Follow-up (M)
	MIDP	ODP	MIDP	ODP	MIDP	ODP	MIDP	ODP	MIDP	ODP	MIDP	ODP	MIDP	ODP	
Kooby^18^	6	24	14.0 ± 8.6	12.3 ± 8.3	NR	NR	13	45	NR	NR	NR	NR	NR	NR	82
Magge^26^	4	4	11 ± 8.9	12 ± 9.6	2	1	25	29	NR	NR	13	13	NR	NR	60
Rehman^16^	1	2	15 ± 6.5	13.5 ± 6.5	NR	NR	4	9	NR	NR	4	9	NR	NR	60
Sharpe^36^	17	127	14.9 ± 10	13.3 ± 9.9	NR	NR	3	69	NR	NR	68	304	NR	NR	NR
Shin^37^	17	13	12 ± 5.5	10 ± 10.5	35	48	55	55	NR	NR	NR	NR	NR	NR	60
Sulpice^40^	NR	NR	NR	NR	NR	NR	NR	NR	NR	NR	NR	NR	2	96	60
Zhang^17^	1	5	9 ± 2.5	8 ± 5	11	16	13	26	12	25	NR	NR	0	1	NR
Stauffer^19^	2	5	25.9 ± 7.2	12.7 ± 7.3	NR	NR	31	18	NR	NR	NR	NR	3	2	60
Bauman^44^	6	6	14.5 ± 1.1	17.5 ± 1.2	10	24	20	29	NR	NR	NR	NR	NR	NR	60
Hilst^12^	122	152	14 ± 10.4	22 ± 12.6	NR	NR	165	159	164	210	NR	NR	19	38	82
Zhang^56^	2	10	11.2 ± 4.6	14.4 ± 5.5	NR	NR	NR	NR	NR	NR	8	31	NR	NR	50

MIDP = Minimally invasive distal pancreatectomy; ODP = Open distal pancreatectomy; NR = Not reported; M = Months; Data shown represents mean ± standard deviation.

**Table 5:** Results of meta-analysis comparing MIDP versus ODP for PDAC.

Outcome of interest	No. of studies	No. of patients	OR/WMD/HR	95%CI	P value	Heterogeneity P value	*I* ^2^
***Primary outcome***							
3-OS	9	3988	1.03	0.89,1.21	0.66	0.67	0%
5-OS	4	3044	0.91	0.65,1.28	0.59	0.35	9%
***Secondary outcomes***							
Operation time	9	1307	5.98	−13.15, 25.11	0.54	<0.00001	88%
Intraoperative blood loss	8	1157	−250.04	−359.69, −140.39	<0.00001	<0.00001	92%
Hospital stay	10	4731	−2.50	−3.36, −1.63	<0.00001	<0.00001	79%
Morbidity	5	2988	0.57	0.46, 0.71	<0.00001	0.69	0%
POPF	7	1152	1.10	0.82, 1.47	0.54	0.56	0%
Mortality	10	4767	0.50	0.31, 0.81	0.005	0.84	0%
Positive surgical margin	10	2076	0.71	0.56, 0.89	0.003	0.39	6%
Lymph nodes harvested	10	2076	0.40	−2.36, 3.16	0.78	<0.00001	95%
Recurrence	4	342	0.74	0.47, 1.18	0.21	0.14	46%
Adjuvant therapy	9	1978	0.94	0.75, 1.18	0.59	0.13	36%
Positive lymphnodes	4	951	0.95	0.69, 1.31	0.76	0.80	0%
Vascular resection	4	3556	0.36	0.22, 0.60	<0.00001	0.30	19%
Perineural and lymfovascular invasion	2	731	0.59	0.44, 0.79	0.0005	0.55	0%

MIDP = Minimally invasive distal pancreatectomy; ODP = Open distal pancreatectomy; OR = Odds ratio; WMD = Weighted Mean Difference; HR = Hazard ratio; PDAC = Pancreatic ductal adenocarcinoma; 3-OS = 3 year overall survival; 5-OS = 5 year overall survival; POPF = Postoperative pancreatic fistula.

### Results of the meta-analysis

#### Primary outcomes

The OS was reported in ten studies^12,16–19,26,37,40,44,56^. However, the OS could not be extracted from the survival curve in a study by Staffer^19^. Finally, nine studies reported 3-year OS, and four studies reported 5-year OS. No statistically significant differences were observed in 3-year OS (HR: 1.03, 95% CI: 0.89, 1.21; *P* = 0.66) or 5-year OS (HR: 0.91, 95% CI: 0.65, 1.28; *P* = 0.59) between the two groups.

#### Secondary outcomes

A positive surgical margin was reported in all included studies. The pooled analysis suggested that MIDP was associated with a lower rate of positive surgical margins (OR: 0.71, 95% CI: 0.56, 0.89; *P* = 0.003). According to the different definitions, the MIDP group also had a lower rate of positive surgical margins for a margin to tumor distance <1 mm^12,16,17^ (OR: 0.66, 95% CI: 0.49, 0.89; *P* = 0.006). However, no significant difference was observed for a margin to tumor distance <0 mm^18,19^ (OR: 0.49, 95% CI: 0.20, 1.20; *P* = 0.12) between the two groups. Only two studies^12,17^ reported perineural and lymphovascular invasion; the MIDP group had less perineural and lymphovascular invasion (OR: 0.59, 95% CI: 0.44, 0.79; *P* = 0.0005) than the ODP group. Positive lymph nodes were reported in four studies^16,26,36,56^; however, no significant difference was found in this outcome between the two groups (OR: 0.95, 95% CI: 0.69, 1.31; *P* = 0.76). The rate of the recurrence was not significantly different (OR: 0.74, 95% CI: 0.47, 1.18; *P* = 0.21) between groups according to the pooled results of 4 studies^17,26,37,59^. Unfortunately, only one study reported multivisceral resection, and no difference (ODP: −32.1% vs MIDP: −38.6%, *P* = 0.66) was found between the two groups. MIDP was associated with less intraoperative blood loss (WMD: −250.03, 95% CI: −359.68, −140.39; *P* < 0.00001), shorter hospital stay (WMD: −2.76, 95% CI: −3.73, −1.78; *P* < 0.00001), and lower morbidity (OR: 0.57, 95% CI: 0.46, 0.71; *P* < 0.00001) and mortality (OR: 0.50; 95% CI: 0.31, 0.81; *P* = 0.005) than ODP. However, no significant difference was observed in operative time (WMD: 5.98, 95% CI: −13.15, 25.11; *P* = 0.54), POPF occurrence (OR: 1.10; 95% CI: 0.82, 1.47; *P* = 0.54), number of lymph nodes harvested (WMD: 0.40, 95% CI: −2.36, 3.16; *P* = 0.78), recurrence (OR: 0.74, 95% CI: 0.47, 1.18; *P* = 0.21) and adjuvant therapy (OR: 0.94; 95% CI: 0.75, 1.18; *P* = 0.59) between the two groups.

#### Sensitivity analysis

A sensitivity analysis was conducted by changing the type of effects model or excluding individual studies from the outcome analysis. The results in operation time were different in the fixed and random effects models. Although high heterogeneity was found for blood loss, this result was presented in both the fixed and random effects models. The heterogeneity was large for hospital stay; however, the heterogeneity was zero when two studies (Sulipice^40^ and Bauman^59^ were excluded.

#### Publication bias

**Figure 2:**
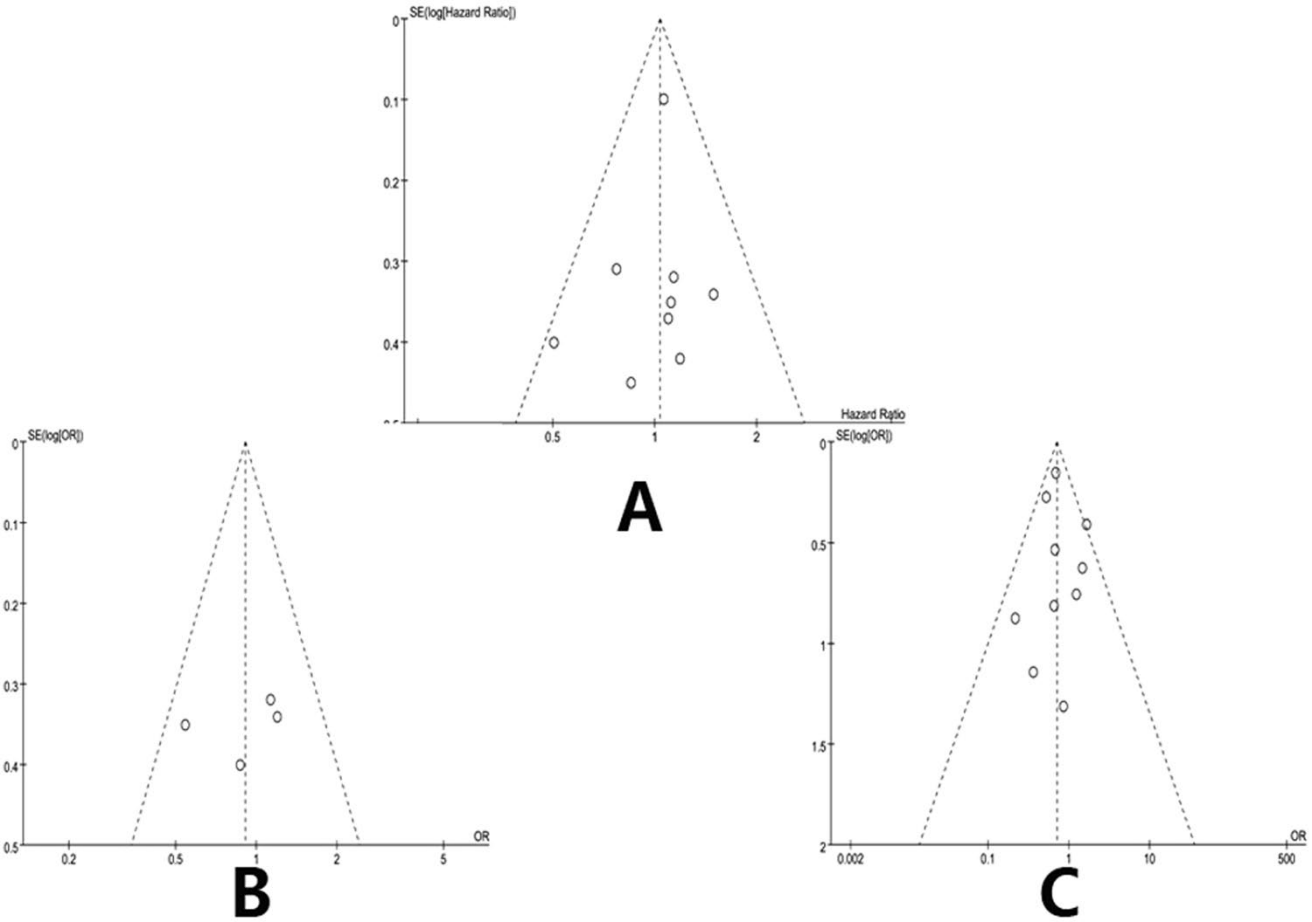
Funnel plot to investigate publication bias. (**A**) Funnel plot based on 3 year overall survival. (**B**) Funnel plot based on 5 year overall survival. (**C**) Funnel plot based on positive surgical margin.

Funnel plots based on the 3-year OS, 5-year OS and positive surgical margins are shown in Fig. 2. No study was outside the limits of the 95% CI; therefore, no evidence of publication bias was present.

## Discussion

MIDP has been performed for more than a decade, and the technical feasibility, safety and clinical benefit have been well documented by many studies comparing it to ODP^34,60,61^. However, some centers still favor adopting a conventional, open approach for PDAC^62,63^.This systematic review and meta-analysis suggested that the OS and oncologic outcomes are not significantly different between the MIDP and ODP groups. Although the pooled results were mainly based on nonrandomized data, the results suggested that MIDP did not adversely affect long-term survival in PDAC patients. Furthermore, the MIDP group also had a lower rate of positive surgical margins and less perineural and lymphovascular invasion. Therefore, MIDP might be feasible and safe regarding long-term survival and oncological outcomes in PDAC patients.

Currently, the rate of long-term survival after distal resection for PDAC patients remains poor. Despite the significant improvement in operative skill for MIDP, the long-term survival is still not different between the two groups of PDAC patients. Radical surgical resection remains the only potentially curative treatment for patients with resectable PDAC^64^. A recent review from a large randomized trial involving resection of PDAC showed positive margin rates ranging from 0% to 83%^65^. The finding in the current report is consistent with those in the published literature^30^. Importantly, the MIDP results in fewer resections with positive margins than ODP. This advantage might be due to the clearer view and more elaborate procedure of MIDP. However, the rate of positive margin resection in present study should be interpreted with caution because margin status was influenced by the use of different definitions and pathologic assessment methods. Furthermore, conclusions may vary by pathologists and institutions due to the absence of standardized pathology assessment and reporting. Moreover, surgeons determine which method is more suitable for the PDAC patients before surgery. Therefore, patients with less extensive cancer may undergo MIDP, and patients with more extensive cancer would undergo ODP^16,36^.

Regarding other oncological outcomes, no significant differences were found for recurrence, lymph nodes harvested and positive lymph nodes. The extent of the lymphadenectomy determined the number of retrieved lymph nodes. According to one report, at least 12 lymph nodes should be evaluated histologically to determine metastatic disease and adequately stage PDAC patients^66^. Moreover, according to the consensus of the International Study Group on Pancreatic Surgery (ISGPS), a standard lymphadenectomy with resection of lymph node stations 10, 11, and 18 is recommended for cancer of the body and tail of the pancreas^67^. In most included studies, the mean number of harvested lymph nodes was greater than 12. In present study, no significant difference was observed in the number of harvested lymph nodes between the two groups. Based on this finding, MIDP is a reasonable procedure for PDAC patients.

No difference was observed in some clinical outcomes. However, MIDP is associated with a steep learning curve. This observation potentially affected several outcomes, including operative time, blood loss and the length of hospital stay^16^. Therefore, the hospital stay and blood loss were decreased in the MIDP group. Moreover, MIDP was associated with significantly lower morbidity and mortality rates than ODP. Our results are consistent with those of many studies. The technical feasibility, safety and clinical benefit have been well confirmed by various matched comparison studies^34,60,61^. Thus, MIDP might be safer with regard to oncological outcomes in PDAC patients.

However, our study has some limitations. First, all of the included studies were retrospective, which could lead to inevitable selection bias toward resection of larger or locally advanced tumors via the open approach, especially in the earlier years of the study period. Second, the follow-up time differed and was relatively short in some studies. Therefore, the long-term survival was difficult to accurately estimate. Third, the definitions of some outcomes were different among studies.

## Conclusion

In summary, the meta-analysis demonstrated that MIDP might be safer with regard to the oncological outcomes of PDAC patients. However, these results need to be confirmed in a future prospective randomized trial.

## Supplementary Information

Supplementary Information
